# Supplementary material for: Mental health in autistic adults: A rapid review of prevalence of psychiatric disorders and umbrella review of the effectiveness of interventions within a neurodiversity informed perspective
Source: PLoS One. 2023 Jul 13;18(7):e0288275. doi: 10.1371/journal.pone.0288275 (PMC10343158; doi:10.1371/journal.pone.0288275)
Supplement: S1 File — (DOCX) [file pone.0288275.s002.docx]

Search Strategy

For the prevalence study: (1) Autism OR Autism Spectrum Disorder OR autistic disorder, AND (2) Prevalence OR frequency, AND (3)
stress, psychological OR burnout, professional OR sexual dysfunctions, psychological OR vaginismus OR anhedonia OR affective symptoms OR *Mental Disorders OR
neurasthenia OR hysteria OR Munchausen syndrome by proxy OR Munchausen syndrome OR fatigue syndrome, chronic OR obsessive behaviour OR compulsive behaviour OR behaviour, addictive OR impulse control disorders OR fire setting behaviour OR gambling OR trichotillomania OR
stress disorders, post-traumatic OR stress disorders, traumatic, acute OR anxiety OR anxiety, castration OR koro OR anxiety, separation OR panic OR exp anti-anxiety agents OR somatoform disorders OR body dysmorphic disorders OR conversion disorders OR hypochondriasis OR
depression OR adjustment disorders OR exp anti depressive agents OR anxiety disorders OR agoraphobia OR neurocirculatory asthenia OR obsessive-compulsive disorder OR obsessive hoarding OR panic disorder OR phobic disorders OR stress disorders, traumatic OR combat disorders OR suicide, attempted OR mood disorders OR affective disorders, psychotic OR bipolar disorder OR cyclothymic disorder OR depressive disorder OR depression, postpartum OR depressive disorder, major OR depressive disorder, treatment-resistant OR dysthymic disorder OR seasonal affective disorder OR neurotic disorders OR
eating disorders OR anorexia nervosa OR binge-eating disorder OR bulimia nervosa OR female athlete triad syndrome OR pica OR hyperphagia OR bulimia OR self-injurious behaviour OR self-mutilation OR suicide OR suicidal ideation.

For the intervention study: (1) Autism OR Autism Spectrum Disorder OR autistic disorder, AND (2) systematic review.
