# Supplementary material for: Mental health in autistic adults: A rapid review of prevalence of psychiatric disorders and umbrella review of the effectiveness of interventions within a neurodiversity informed perspective
Source: PLoS One. 2023 Jul 13;18(7):e0288275. doi: 10.1371/journal.pone.0288275 (PMC10343158; doi:10.1371/journal.pone.0288275)
Supplement: S2 File — (DOCX) [file pone.0288275.s003.docx]

**Studies excluded from Prevalence Review**

Arwert, T. G., & Sizoo, B. B. (2020). Self-reported suicidality in male and female adults with autism spectrum disorders: Rumination and self-esteem. *Journal of Autism and Developmental Disorders, 50*(10), 3598-3605. doi:10.1007/s10803-020-04372-z

Bakken, T. L., Helverschou, S. B., Eilertsen, D. E., Heggelund, T., Myrbakk, E., & Martinsen, H. (2010). Psychiatric disorders in adolescents and adults with autism and intellectual disability: A representative study in one county in Norway. *Research in Developmental Disabilities, 31*(6), 1669-1677. doi:10.1016/j.ridd.2010.04.009

Baudewijns, L., Ronsse, E., Verstraete, V., Sabbe, B., Morrens, M., & Bertelli, M. O. (2018). Problem behaviours and major depressive disorder in adults with intellectual disability and autism. *Psychiatry Research, 270*, 769-774. doi:10.1016/j.psychres.2018.10.039

Bedford, S. A., Hunsche, M. C., & Kerns, C. M. (2020). Co-occurrence, assessment and treatment of obsessive compulsive disorder in children and adults with autism spectrum disorder. *Current Psychiatry Reports, 22*(10), 53. doi:10.1007/s11920-020-01176-x

Breen, J., & Hare, D. J. (2017). The nature and prevalence of catatonic symptoms in young people with autism. *Journal of Intellectual Disability Research: JIDR, 61*(6), 580-593. doi:10.1111/jir.12362

Dell'Osso, L., Carpita, B., Muti, D., Morelli, V., Salarpi, G., Salerni, A., . . . Maj, M. (2019). Mood symptoms and suicidality across the autism spectrum. *Comprehensive Psychiatry, 91*, 34-38. doi:10.1016/j.comppsych.2019.03.004

Doupnik, S. K., Henry, M. K., Bae, H., Litman, J., Turner, S., Scharko, A. M., & Feudtner, C. (2017). Mental health conditions and symptoms in pediatric hospitalizations: A single-center point prevalence study. *Academic Pediatrics, 17*(2), 184-190. doi:10.1016/j.acap.2016.08.009

Folch, A., Cortés, M. J., Salvador-Carulla, L., Vicens, P., Irazábal, M., Muñoz, S., . . . Martínez-Leal, R. (2018). Risk factors and topographies for self-injurious behaviour in a sample of adults with intellectual developmental disorders. *Journal of Intellectual Disability Research: JIDR, 62*(12), 1018-1029. doi:10.1111/jir.12487

Hannon, G., & Taylor, E. P. (2013). Suicidal behaviour in adolescents and young adults with ASD: Findings from a systematic review. *Clinical Psychology Review, 33*(8), 1197-1204. doi:10.1016/j.cpr.2013.10.003

Horowitz, L. M., Thurm, A., Farmer, C., Mazefsky, C., Lanzillo, E., Bridge, J. A., . . . Siegel, M. (2018). Talking about death or suicide: Prevalence and clinical correlates in youth with autism spectrum disorder in the psychiatric inpatient setting. *Journal of Autism and Developmental Disorders, 48*(11), 3702-3710. doi:10.1007/s10803-017-3180-7

Hryniewiecka-Jaworska, A., Foden, E., Kerr, M., Felce, D., & Clarke, A. (2016). Prevalence and associated features of depression in women with Rett syndrome. *Journal of Intellectual Disability Research: JIDR, 60*(6), 564-570. doi:10.1111/jir.12270

Joshi, G., Wozniak, J., Fitzgerald, M., Faraone, S., Fried, R., Galdo, M., . . . Biederman, J. (2018). High risk for severe emotional dysregulation in psychiatrically referred youth with autism spectrum disorder: A controlled study. *Journal of Autism and Developmental Disorders, 48*(9), 3101-3115. doi:10.1007/s10803-018-3542-9

Kiani, R., Tyrer, F., Hodgson, A., Berkin, N., & Bhaumik, S. (2013). Urban-rural differences in the nature and prevalence of mental ill-health in adults with intellectual disabilities. *Journal of Intellectual Disability Research: JIDR, 57*(2), 119-127. doi:10.1111/j.1365-2788.2011.01523.x

Kreiser, N. L., & White, S. W. (2015). ASD traits and co-occurring psychopathology: The moderating role of gender. *Journal of Autism and Developmental Disorders, 45*(12), 3932-3938. doi:10.1007/s10803-015-2580-9

Leader, G., Grennan, S., Chen, J. L., & Mannion, A. (2018). An investigation of gelotophobia in individuals with a diagnosis of high-functioning autism spectrum disorder. *Journal of Autism and Developmental Disorders, 48*(12), 4155-4166. doi:10.1007/s10803-018-3661-3

Licence, L., Oliver, C., Moss, J., & Richards, C. (2020). Prevalence and risk-markers of self-harm in autistic children and adults. *Journal of Autism and Developmental Disorders, 50*(10), 3561-3574. doi:10.1007/s10803-019-04260-1

Lundqvist, L. (2013). Prevalence and risk markers of behavior problems among adults with intellectual disabilities: A total population study in Orebro County, Sweden. *Research in Developmental Disabilities, 34*(4), 1346-1356. doi:10.1016/j.ridd.2013.01.010

Nah, Y., Brewer, N., Young, R. L., & Flower, R. (2018). Brief report: Screening adults with autism spectrum disorder for anxiety and depression. *Journal of Autism and Developmental Disorders, 48*(5), 1841-1846. doi:10.1007/s10803-017-3427-3

Nylander, L., Holmqvist, M., Gustafson, L., & Gillberg, C. (2013). Attention-deficit/hyperactivity disorder (ADHD) and autism spectrum disorder (ASD) in adult psychiatry. A 20-year register study. *Nordic Journal of Psychiatry, 67*(5), 344-350. doi:10.3109/08039488.2012.748824

Paquette-Smith, M., Weiss, J., & Lunsky, Y. (2014). History of suicide attempts in adults with Asperger syndrome. *Crisis, 35*(4), 273-277. doi:10.1027/0227-5910/a000263

Postorino, V., Kerns, C. M., Vivanti, G., Bradshaw, J., Siracusano, M., & Mazzone, L. (2017). Anxiety disorders and obsessive-compulsive disorder in individuals with autism spectrum disorder. *Current Psychiatry Reports, 19*(12), 92. doi:10.1007/s11920-017-0846-y

Raja, M., & Azzoni, A. (2010). Autistic spectrum disorders and schizophrenia in the adult psychiatric setting: Diagnosis and comorbidity. *Psychiatria Danubina, 22*(4), 514-521.

Rattaz, C., Michelon, C., Munir, K., & Baghdadli, A. (2018). Challenging behaviours at early adulthood in autism spectrum disorders: Topography, risk factors and evolution. *Journal of Intellectual Disability Research: JIDR, 62*(7), 637-649. doi:10.1111/jir.12503

Strauss, P., Cook, A., Watson, V., Winter, S., Whitehouse, A., Albrecht, N., . . . Lin, A. (2021). Mental health difficulties among trans and gender diverse young people with an autism spectrum disorder (ASD): Findings from trans pathways. *Journal of Psychiatric Research, 137*, 360-367. doi:10.1016/j.jpsychires.2021.03.005

Tebartz van Elst, L., Pick, M., Biscaldi, M., Fangmeier, T., & Riedel, A. (2013). High-functioning autism spectrum disorder as a basic disorder in adult psychiatry and psychotherapy: Psychopathological presentation, clinical relevance and therapeutic concepts. *European Archives of Psychiatry and Clinical Neuroscience, 263 Suppl 2*, S189-S196. doi:10.1007/s00406-013-0459-3

Tromans, S., Yao, G. L., Kiani, R., Alexander, R., Al-Uzri, M., & Brugha, T. (2019). Study protocol: An investigation of the prevalence of autism among adults admitted to acute mental health wards: A cross-sectional pilot study. *BMJ Open, 9*(12), e033169. doi:10.1136/bmjopen-2019-033169

Turygin, N. C., Matson, J. L., Adams, H. L., & Williams, L. W. (2014). Co-occurring disorder clusters in adults with mild and moderate intellectual disability in residential treatment settings. *Research in Developmental Disabilities, 35*(11), 3156-3161. doi:10.1016/j.ridd.2014.07.039

Vannucchi, G., Masi, G., Toni, C., Dell'Osso, L., Erfurth, A., & Perugi, G. (2014). Bipolar disorder in adults with Asperger's syndrome: A systematic review. *Journal of Affective Disorders, 168*, 151-160. doi:10.1016/j.jad.2014.06.042

White, S. W., Ollendick, T. H., & Bray, B. C. (2011). College students on the autism spectrum: Prevalence and associated problems. *Autism: The International Journal of Research and Practice, 15*(6), 683-701. doi:10.1177/1362361310393363

Zukerman, G., Yahav, G., & Ben-Itzchak, E. (2019). Increased psychiatric symptoms in university students with autism spectrum disorder are associated with reduced adaptive behavior. *Psychiatry Research, 273*, 732-738. doi:10.1016/j.psychres.2019.01.098

**Studies excluded from the Rapid Review of Reviews of Intervention Studies**

Akers, J. S., Davis, T. N., Gerow, S., & Avery, S. (2020). Decreasing motor stereotypy in individuals with autism spectrum disorder: A systematic review. *Research in Autism Spectrum Disorders, 77* doi:10.1016/j.rasd.2020.101611

Alallawi, B., Hastings, R. P., & Gray, G. (2020). A systematic scoping review of social, educational, and psychological research on individuals with autism spectrum disorder and their family members in Arab countries and cultures. *Review Journal of Autism and Developmental Disorders, 7*(4), 364-382. doi:10.1007/s40489-020-00198-8

Allely, C. S. (2018). A systematic PRISMA review of individuals with autism spectrum disorder in secure psychiatric care: Prevalence, treatment, risk assessment and other clinical considerations. *Journal of Criminal Psychology, 8*(1), 58-79. doi:10.1108/JCP-06-2017-0028

Ameis, S. H., Kassee, C., Corbett-Dick, P., Cole, L., Dadhwal, S., Lai, M. -., . . . Correll, C. U. (2018). Systematic review and guide to management of core and psychiatric symptoms in youth with autism. *Acta Psychiatrica Scandinavica, 138*(5), 379-400. doi:10.1111/acps.12918

Anderson, A. H., Stephenson, J., Carter, M., & Carlon, S. (2019). A systematic literature review of empirical research on postsecondary students with autism spectrum disorder. *Journal of Autism and Developmental Disorders, 49*(4), 1531-1558. doi:10.1007/s10803-018-3840-2

Ansari, F., Pourjafar, H., Tabrizi, A., & Homayouni, A. (2020). The effects of probiotics and prebiotics on mental disorders: A review on depression, anxiety, Alzheimer, and autism spectrum disorders. *Current Pharmaceutical Biotechnology, 21*(7), 555-565. doi:10.2174/1389201021666200107113812

Aresti-Bartolome, N., & Garcia-Zapirain, B. (2014). Technologies as support tools for persons with autistic spectrum disorder: A systematic review. *International Journal of Environmental Research and Public Health, 11*(8), 7767-7802. doi:10.3390/ijerph110807767

Arnevik, E. A., & Helverschou, S. B. (2016). Autism spectrum disorder and co-occurring substance use disorder - A systematic review. *Substance Abuse: Research and Treatment, 10*, 69-75. doi:10.4137/SART.S39921

Aye, S. Z., Ni, H., Sein, H. H., Mon, S. T., Zheng, Q., & Wong, Y. K. (2021). The effectiveness and adverse effects of D‐cycloserine compared with placebo on social and communication skills in individuals with autism spectrum disorder. *Cochrane Database of Systematic Reviews,* (2) doi:10.1002/14651858.CD013457.pub2

Banas, K., & Sawchuk, B. (2020). Clonidine as a treatment of behavioural disturbances in autism spectrum disorder: A systematic literature review. *Journal of the Canadian Academy of Child and Adolescent Psychiatry, 29*(2), 110-120.

Barnard, L., Young, A. H., Pearson, J., Geddes, J., & O'Brien, G. (2002). A systematic review of the use of atypical antipsychotics in autism. *Journal of Psychopharmacology, 16*(1), 93-101. doi:10.1177/026988110201600113

Boster, J. B., Spitzley, A. M., Castle, T. W., Jewell, A. R., Corso, C. L., & McCarthy, J. W. (2021). Music improves social and participation outcomes for individuals with communication disorders: A systematic review. *Journal of Music Therapy, 58*(1), 12-42. doi:10.1093/jmt/thaa015

Broadstock, M., Doughty, C., & Eggleston, M. (2007). Systematic review of the effectiveness of pharmacological treatments for adolescents and adults with autism spectrum disorder. *Autism, 11*(4), 335-348. doi:10.1177/1362361307078132

Chahin, S. S., Apple, R. W., Kuo, K. H., & Dickson, C. A. (2020). Autism spectrum disorder: Psychological and functional assessment, and behavioral treatment approaches. *Translational Pediatrics, 9*, S66-S75. doi:10.21037/TP.2019.11.06

Chan, J. M., Lang, R., Rispoli, M., O'Reilly, M., Sigafoos, J., & Cole, H. (2009). Use of peer-mediated interventions in the treatment of autism spectrum disorders: A systematic review. *Research in Autism Spectrum Disorders, 3*(4), 876-889. doi:10.1016/j.rasd.2009.04.003

Chandroo, R., Strnadová, I., & Cumming, T. M. (2018). A systematic review of the involvement of students with autism spectrum disorder in the transition planning process: Need for voice and empowerment. *Research in Developmental Disabilities, 83*, 8-17. doi:10.1016/j.ridd.2018.07.011

Chen, B. B., & Yakubova, G. (2021). Evaluating the effects of video-based intervention to teach vocational skills to transition-age youth with autism spectrum disorder: An evidence-based systematic review. *Review Journal of Autism and Developmental Disorders,* doi:10.1007/s40489-021-00282-7

Chia, G. L. C., Anderson, A., & McLean, L. A. (2018). Use of technology to support self-management in individuals with autism: Systematic review. *Review Journal of Autism and Developmental Disorders, 5*(2), 142-155. doi:10.1007/s40489-018-0129-5

Cortese, S., Castelnau, P., Morcillo, C., Roux, S., & Bonnet-Brilhault, F. (2012). Psychostimulants for ADHD-like symptoms in individuals with autism spectrum disorders. *Expert Review of Neurotherapeutics, 12*(4), 461-473. doi:10.1586/ern.12.23

Davis, T. N., O'Reilly, M., Kang, S., Lang, R., Rispoli, M., Sigafoos, J., . . . Mulloy, A. (2013). Chelation treatment for autism spectrum disorders: A systematic review. *Research in Autism Spectrum Disorders, 7*(1), 49-55. doi: 10.1016/j.rasd.2012.06.005

Deepmala, Slattery, J., Kumar, N., Delhey, L., Berk, M., Dean, O., . . . Frye, R. (2015). Clinical trials of N-acetylcysteine in psychiatry and neurology: A systematic review. *Neuroscience and Biobehavioral Reviews, 55*, 294-321. doi:10.1016/j.neubiorev.2015.04.015

DeJong, H., Bunton, P., & Hare, D. J. (2014). A systematic review of interventions used to treat catatonic symptoms in people with autistic spectrum disorders. *Journal of Autism and Developmental Disorders, 44*(9), 2127-2136. doi:10.1007/s10803-014-2085-y

Den Brok, W. L. J. E., & Sterkenburg, P. S. (2015). Self-controlled technologies to support skill attainment in persons with an autism spectrum disorder and/or an intellectual disability: A systematic literature review. *Disability and Rehabilitation: Assistive Technology, 10*(1), 1-10. doi:10.3109/17483107.2014.921248

Denizli-Gulboy, H., Genc-Tosun, D., & Gulboy, E. (2021). Evaluating augmented reality as evidence-based practice for individuals with autism spectrum disorder: A meta-analysis of single-case design studies. *International Journal of Developmental Disabilities,* doi:10.1080/20473869.2021.1972741

Desideri, L., Di Santantonio, A., Varrucciu, N., Bonsi, I., & Di Sarro, R. (2020). Assistive technology for cognition to support executive functions in autism: A scoping review. *Advances in Neurodevelopmental Disorders, 4*(4), 330-343. doi:10.1007/s41252-020-00163-w

Dinnissen, M., Dietrich, A., Van Den Hoofdakker, B. J., & Hoekstra, P. J. (2015). Clinical and pharmacokinetic evaluation of risperidone for the management of autism spectrum disorder. *Expert Opinion on Drug Metabolism and Toxicology, 11*(1), 111-124. doi:10.1517/17425255.2015.981151

Doherty, A. J., Atherton, H., Boland, P., Hastings, R., Hives, L., Hood, K., . . . Chauhan, U. (2020). Barriers and facilitators to primary health care for people with intellectual disabilities and/or autism: An integrative review. *BJGP Open, 4*(3) doi:10.3399/bjgpopen20X101030

Dove, D., Warren, Z., McPheeters, M. L., Taylor, J. L., Sathe, N. A., & Veenstra-VanderWeele, J. (2012). Medications for adolescents and young adults with autism spectrum disorders: A systematic review. *Pediatrics, 130*(4), 717-726. doi:10.1542/peds.2012-0683

Fragala-Pinkham, M., Ball, A. L., & Jeffries, L. M. (2021). Efficacy of lower extremity cycling interventions for youth with intellectual disabilities: A systematic review. *Physical & Occupational Therapy in Pediatrics, 41*(4), 410-428. doi:10.1080/01942638.2020.1862384

Frogley, C., Taylor, D., Dickens, G., & Picchioni, M. (2012). A systematic review of the evidence of clozapine's anti-aggressive effects. *The International Journal of Neuropsychopharmacology, 15*(9), 1351-1371. doi:10.1017/S146114571100201X

Frye, R. E., Rossignol, D., Casanova, M. F., Martin, V., Brown, G. L., Edelson, S., . . . Adams, J. B. (2013). A review of traditional and novel treatments for seizures in autism spectrum disorder: Findings from a systematic review and expert panel. *Frontiers in Public Health, 1* doi:10.3389/fpubh.2013.00031

Garcia, Y., Keller-Collins, A., Andrews, M., Kurumiya, Y., Imlay, K., Umphrey, B., & Foster, E. (2021). Systematic review of acceptance and commitment therapy in individuals with neurodevelopmental disorders, caregivers, and staff. *Behavior Modification,* doi:10.1177/01454455211027301

Gates, J. A., Kang, E., & Lerner, M. D. (2017). Efficacy of group social skills interventions for youth with autism spectrum disorder: A systematic review and meta-analysis. *Clinical Psychology Review, 52*, 164-181. doi:10.1016/j.cpr.2017.01.006

Gelbar, N. W., Smith, I., & Reichow, B. (2014). Systematic review of articles describing experience and supports of individuals with autism enrolled in college and university programs. *Journal of Autism and Developmental Disorders, 44*(10), 2593-2601. doi:10.1007/s10803-014-2135-5

Guénolé, F., Godbout, R., Nicolas, A., Franco, P., Claustrat, B., & Baleyte, J. (2011). Melatonin for disordered sleep in individuals with autism spectrum disorders: Systematic review and discussion. *Sleep Medicine Reviews, 15*(6), 379-387. doi:10.1016/j.smrv.2011.02.001

Healy, S., Nacario, A., Braithwaite, R. E., & Hopper, C. (2018). The effect of physical activity interventions on youth with autism spectrum disorder: A meta-analysis. *Autism Research, 11*(6), 818-833. doi:10.1002/aur.1955

Healy, S., Pacanowski, C. R., & Williams, E. (2019). Weight management interventions for youth with autism spectrum disorder: A systematic review. *International Journal of Obesity, 43*(1) doi:10.1038/s41366-018-0233-8

Heyvaert, M., Saenen, L., Campbell, J. M., Maes, B., & Onghena, P. (2014). Efficacy of behavioral interventions for reducing problem behavior in persons with autism: An updated quantitative synthesis of single-subject research. *Research in Developmental Disabilities, 35*(10), 2463-2476. doi:10.1016/j.ridd.2014.06.017

Holbrook, S., & Israelsen, M. (2020). Speech prosody interventions for persons with autism spectrum disorders: A systematic review. *American Journal of Speech-Language Pathology, 29*(4), 2189-2205. doi:10.1044/2020_AJSLP-19-00127

Holyfield, C., Drager, K. D. R., Kremkow, J. M. D., & Light, J. (2017). Systematic review of AAC intervention research for adolescents and adults with autism spectrum disorder. *Augmentative and Alternative Communication (Baltimore, Md.: 1985), 33*(4), 201-212. doi:10.1080/07434618.2017.1370495

Hong, E. R., Gong, L. -., Ninci, J., Morin, K., Davis, J. L., Kawaminami, S., . . . Noro, F. (2017). A meta-analysis of single-case research on the use of tablet-mediated interventions for persons with ASD. *Research in Developmental Disabilities, 70*, 198-214. doi:10.1016/j.ridd.2017.09.013

Hong, E. R., Ganz, J. B., Mason, R., Morin, K., Davis, J. L., Ninci, J., . . . Gilliland, W. D. (2016). The effects of video modeling in teaching functional living skills to persons with ASD: A meta-analysis of single-case studies. *Research in Developmental Disabilities, 57*, 158-169. doi:10.1016/j.ridd.2016.07.001

Hume, K., Steinbrenner, J. R., Odom, S. L., Morin, K. L., Nowell, S. W., Tomaszewski, B., . . . Savage, M. N. (2021). Evidence-based practices for children, youth, and young adults with autism: Third generation review. *Journal of Autism and Developmental Disorders, 51*(11), 4013-4032. doi:10.1007/s10803-020-04844-2

Hutchinson, N., & Bodicoat, A. (2015). The effectiveness of intensive interaction, A systematic literature review. *Journal of Applied Research in Intellectual Disabilities: JARID, 28*(6), 437-454. doi:10.1111/jar.12138

Iasevoli, F., Barone, A., Buonaguro, E. F., Vellucci, L., & de Bartolomeis, A. (2020). Safety and tolerability of antipsychotic agents in neurodevelopmental disorders: A systematic review. *Expert Opinion on Drug Safety, 19*(11), 1419-1444. doi:10.1080/14740338.2020.1820985

Jesner, O. S., Aref‐Adib, M., & Coren, E. (2007). Risperidone for autism spectrum disorder. *Cochrane Database of Systematic Reviews,* (1) doi:10.1002/14651858.CD005040.pub2

Karami, B., Koushki, R., Arabgol, F., Rahmani, M., & Vahabie, A. -. (2021). Effectiveness of Virtual/Augmented Reality–Based therapeutic interventions on individuals with autism spectrum disorder: A comprehensive meta-analysis. *Frontiers in Psychiatry, 12* doi:10.3389/fpsyt.2021.665326

Kirby, A. V., Baranek, G. T., & Fox, L. (2016). Longitudinal predictors of outcomes for adults with autism spectrum disorder: Systematic review. *OTJR Occupation, Participation and Health, 36*(2), 55-64. doi:10.1177/1539449216650182

Kolevzon, A., Mathewson, K. A., & Hollander, E. (2006). Selective serotonin reuptake inhibitors in autism: A review of efficacy and tolerability. *Journal of Clinical Psychiatry, 67*(3), 407-414. doi:10.4088/JCP.v67n0311

Koumpouros, Y., & Kafazis, T. (2019). Wearables and mobile technologies in autism spectrum disorder interventions: A systematic literature review. *Research in Autism Spectrum Disorders, 66* doi:10.1016/j.rasd.2019.05.005

Kulawiak, P. R. (2021). Academic benefits of wearing noise-cancelling headphones during class for typically developing students and students with special needs: A scoping review. *Cogent Education, 8*(1) doi:10.1080/2331186X.2021.1957530

Lang, R., Mahoney, R., El Zein, F., Delaune, E., & Amidon, M. (2011). Evidence to practice: Treatment of anxiety in individuals with autism spectrum disorders. *Neuropsychiatric Disease and Treatment, 7*(1), 27-30. doi:10.2147/NDT.S10327

Lang, R., Regester, A., Lauderdale, S., Ashbaugh, K., & Haring, A. (2010). Treatment of anxiety in autism spectrum disorders using cognitive behaviour therapy: A systematic review. *Developmental Neurorehabilitation, 13*(1), 53-63. doi:10.3109/17518420903236288

Ledford, J. R., Whiteside, E., & Severini, K. E. (2018). A systematic review of interventions for feeding-related behaviors for individuals with autism spectrum disorders. *Research in Autism Spectrum Disorders, 52*, 69-80. doi:10.1016/j.rasd.2018.04.008

Leung, P. W. S., Li, S. X., Tsang, C. S. O., Chow, B. L. C., & Wong, W. C. W. (2021). Effectiveness of using mobile technology to improve cognitive and social skills among individuals with autism spectrum disorder: Systematic literature review. *JMIR Mental Health, 8*(9) doi:10.2196/20892

Liao, C. -., Ganz, J. B., Vannest, K. J., Wattanawongwan, S., Pierson, L. M., Yllades, V., & Li, Y. -. (2021). Caregiver involvement in communication intervention for culturally and linguistically diverse families with individuals with ASD and IDD: A systematic review of cross-cultural research. *Review Journal of Autism and Developmental Disorders,* doi:10.1007/s40489-021-00288-1

Marcotte, J., Grandisson, M., Piquemal, C., Boucher, A., Rheault, M., & Milot, É. (2020). Supporting independence at home of people with autism spectrum disorder: Literature review. *Canadian Journal of Occupational Therapy. Revue Canadienne d'Ergotherapie, 87*(2), 100-116. doi:10.1177/0008417419890179

Marí-Bauset, S., Zazpe, I., Mari-Sanchis, A., Llopis-González, A., & Morales-Suárez-Varela, M. (2014). Evidence of the gluten-free and casein-free diet in autism spectrum disorders: A systematic review. *Journal of Child Neurology, 29*(12), 1718-1727. doi:10.1177/0883073814531330

Marino, L., & Lilienfeld, S. O. (2021). Third time's the charm or three strikes you're out? an updated review of the efficacy of dolphin-assisted therapy for autism and developmental disabilities. *Journal of Clinical Psychology, 77*(6), 1265-1279. doi:10.1002/jclp.23110

McGonigle, J. J., Venkat, A., Beresford, C., Campbell, T. P., & Gabriels, R. L. (2014). Management of agitation in individuals with autism spectrum disorders in the emergency department. *Child and Adolescent Psychiatric Clinics of North America, 23*(1), 83-95. doi:10.1016/j.chc.2013.08.003

McGuinness, G., & Kim, Y. (2020). Sulforaphane treatment for autism spectrum disorder: A systematic review. *EXCLI Journal, 19*, 892-903. doi:10.17179/excli2020-2487

Menezes, M., Robinson, L., Sanchez, M. J., & Cook, B. (2018). Depression in youth with autism spectrum disorders: A systematic review of studies published between 2012 and 2016. *Review Journal of Autism and Developmental Disorders, 5*(4), 370-389. doi:10.1007/s40489-018-0146-4

Mirzaei, S. S., Pakdaman, S., Alizadeh, E., & Pouretemad, H. (2020). A systematic review of program circumstances in training social skills to adolescents with high-functioning autism. *International Journal of Developmental Disabilities,* doi:10.1080/20473869.2020.1748802

Miyahara, M. (2013). Meta review of systematic and meta-analytic reviews on movement differences, effect of movement-based interventions, and the underlying neural mechanisms in autism spectrum disorder. *Frontiers in Integrative Neuroscience,* doi:10.3389/fnint.2013.00016

Morán, M. ª., Hagiwara, M., Raley, S. K., Alsaeed, A. H., Shogren, K. A., Qian, X., . . . Alcedo, M. ª. (2020). Self-determination of students with autism spectrum disorder: A systematic review. *Journal of Developmental and Physical Disabilities,* doi:10.1007/s10882-020-09779-1

Morris, R., Greenblatt, A., & Saini, M. (2021). Working beyond capacity: A qualitative review of research on healthcare providers’ experiences with autistic individuals. *Review Journal of Autism and Developmental Disorders,* doi:10.1007/s40489-021-00283-6

Muharib, R., Walker, V. L., Alresheed, F., & Gerow, S. (2021). Effects of multiple schedules of reinforcement on appropriate communication and challenging behaviors: A meta-analysis. *Journal of Autism and Developmental Disorders, 51*(2), 613-631. doi:10.1007/s10803-020-04569-2

Myles, B. S. (2003). Behavioral forms of stress management for individuals with Asperger syndrome. *Child and Adolescent Psychiatric Clinics of North America, 12*(1), 123-141. doi:10.1016/S1056-4993(02)00048-2

Ng, Q. X., Loke, W., Venkatanarayanan, N., Lim, D. Y., Soh, A. Y. S., & Yeo, W. S. (2019). A systematic review of the role of prebiotics and probiotics in autism spectrum disorders. *Medicina (Lithuania), 55*(5) doi:10.3390/medicina55050129

Nieforth, L. O., Schwichtenberg, A. J., & O’Haire, M. E. (2021). Animal-assisted interventions for autism spectrum disorder: A systematic review of the literature from 2016 to 2020. *Review Journal of Autism and Developmental Disorders,* doi:10.1007/s40489-021-00291-6

O’Haire, M. E. (2017). Research on animal-assisted intervention and autism spectrum disorder, 2012–2015. *Applied Developmental Science, 21*(3), 200-216. doi:10.1080/10888691.2016.1243988

Ospina, M. B., Seida, J. K., Clark, B., Karkhaneh, M., Hartling, L., Tjosvold, L., . . . Smith, V. (2008). Behavioural and developmental interventions for autism spectrum disorder: A clinical systematic review. *Plos One, 3*(11) doi:10.1371/journal.pone.0003755

Palmen, A., Didden, R., & Lang, R. (2012). A systematic review of behavioral intervention research on adaptive skill building in high-functioning young adults with autism spectrum disorder. *Research in Autism Spectrum Disorders, 6*(2), 602-617. doi:10.1016/j.rasd.2011.10.001

Pioggia, G., Tonacci, A., Tartarisco, G., Billeci, L., Muratori, F., Ruta, L., & Gangemi, S. (2014). Autism and lack of D3 vitamin: A systematic review. *Research in Autism Spectrum Disorders, 8*(12), 1685-1698. doi:10.1016/j.rasd.2014.09.003

Qi, C. H., Barton, E. E., Collier, M., & Lin, Y. -. (2018). A systematic review of single-case research studies on using video modeling interventions to improve social communication skills for individuals with autism spectrum disorder. *Focus on Autism and Other Developmental Disabilities, 33*(4), 249-257. doi:10.1177/1088357617741282

Ramdoss, S., Mulloy, A., Lang, R., O'Reilly, M., Sigafoos, J., Lancioni, G., . . . El Zein, F. (2011). Use of computer-based interventions to improve literacy skills in students with autism spectrum disorders: A systematic review. *Research in Autism Spectrum Disorders, 5*(4), 1306-1318. doi:10.1016/j.rasd.2011.03.004

Raulston, T., Carnett, A., Lang, R., Tostanoski, A., Lee, A., Machalicek, W., . . . Lancioni, G. E. (2013). Teaching individuals with autism spectrum disorder to ask questions: A systematic review. *Research in Autism Spectrum Disorders, 7*(7), 866-878. doi:10.1016/j.rasd.2013.03.008

Ressel, M., Thompson, B., Poulin, M. -., Normand, C. L., Fisher, M. H., Couture, G., & Iarocci, G. (2020). Systematic review of risk and protective factors associated with substance use and abuse in individuals with autism spectrum disorders. *Autism, 24*(4), 899-918. doi:10.1177/1362361320910963

Rogers, K., & Zeni, M. B. (2015). Systematic review of medical home models to promote transitions to primary adult health care for adolescents living with autism spectrum disorder. *Worldviews on Evidence-Based Nursing, 12*(2), 98-107. doi:10.1111/wvn.12085

Rossignol, D. A., & Frye, R. E. (2014). The use of medications approved for Alzheimer's disease in autism spectrum disorder: A systematic review. *Frontiers in Pediatrics, 2* doi:10.3389/fped.2014.00087

Rumball, F. (2019). A systematic review of the assessment and treatment of posttraumatic stress disorder in individuals with autism spectrum disorders. *Review Journal of Autism and Developmental Disorders, 6*(3), 294-324. doi:10.1007/s40489-018-0133-9

Saxena, V., Chacko, G., & Saxena, U. (2021). Systematic review of the effectiveness of homoeopathy in the treatment of autism spectrum disorder. *Clinical Archives of Communication Disorders, 6*(1), 1-11. doi:10.21849/CACD.2021.00339

Schlosser, R. W., Hemsley, B., Shane, H., Todd, J., Lang, R., Lilienfeld, S. O., . . . Odom, S. (2019). Rapid prompting method and autism spectrum disorder: Systematic review exposes lack of evidence. *Review Journal of Autism and Developmental Disorders, 6*(4), 403-412. doi:10.1007/s40489-019-00175-w

Sefen, J. A. N., Al-Salmi, S., Shaikh, Z., AlMulhem, J. T., Rajab, E., & Fredericks, S. (2020). Beneficial use and potential effectiveness of physical activity in managing autism spectrum disorder. *Frontiers in Behavioral Neuroscience, 14* doi:10.3389/fnbeh.2020.587560

Seida, J. K., Ospina, M. B., Karkhaneh, M., Hartling, L., Smith, V., & Clark, B. (2009). Systematic reviews of psychosocial interventions for autism: An umbrella review. *Developmental Medicine and Child Neurology, 51*(2), 95-104. doi:10.1111/j.1469-8749.2008.03211.x

Shaw, A., Do, T. N. T., Harrison, L., Marczak, M., Dimitriou, D., & Joyce, A. (2021). Sleep and cognition in people with autism spectrum condition: A systematic literature review. *Review Journal of Autism and Developmental Disorders,* doi:10.1007/s40489-021-00266-7

Shea, K., Sellers, T. P., Brodhead, M. T., Kipfmiller, K. J., & Sipila-Thomas, E. (2019). A review of mand frame training procedures for individuals with autism. *European Journal of Behavior Analysis, 20*(2), 230-243. doi:10.1080/15021149.2019.1661708

Shire, S. Y., & Kasari, C. (2014). Train the trainer effectiveness trials of behavioral intervention for individuals with autism: A systematic review. *American Journal on Intellectual and Developmental Disabilities, 119*(5), 436-451+488+490. doi:10.1352/1944-7558-119.5.436

Silva, G. M., Souto, J. J. D. S., Fernandes, T. P., Bolis, I., & Santos, N. A. (2021). Interventions with serious games and entertainment games in autism spectrum disorder: A systematic review. *Developmental Neuropsychology,* doi:10.1080/87565641.2021.1981905

Sinha, Y., Silove, N., Wheeler, D., & Williams, K. (2006). Auditory integration training and other sound therapies for autism spectrum disorders: A systematic review. *Archives of Disease in Childhood, 91*(12), 1018-1022. doi:10.1136/adc.2006.094649

Sochocky, N., & Milin, R. (2013). Second generation antipsychotics in Asperger's disorder and high functioning autism: A systematic review of the literature and effectiveness of meta-analysis. *Current Clinical Pharmacology, 8*(4), 370-379. doi:10.2174/15748847113086660073

Srinivasan, S. M., Cavagnino, D. T., & Bhat, A. N. (2018). Effects of equine therapy on individuals with autism spectrum disorder: A systematic review. *Review Journal of Autism and Developmental Disorders, 5*(2), 156-175. doi:10.1007/s40489-018-0130-z

Sturmey, P. (2012). Treatment of psychopathology in people with intellectual and other disabilities. *Canadian Journal of Psychiatry. Revue Canadienne De Psychiatrie, 57*(10), 593-600. doi:10.1177/070674371205701003

Tan, B. W. Z., Pooley, J. A., & Speelman, C. P. (2016). A meta-analytic review of the efficacy of physical exercise interventions on cognition in individuals with autism spectrum disorder and ADHD. *Journal of Autism and Developmental Disorders, 46*(9), 3126-3143. doi:10.1007/s10803-016-2854-x

Tang, J. S. Y., Chen, N. T. M., Falkmer, M., Bӧlte, S., & Girdler, S. (2019). A systematic review and meta-analysis of social emotional computer based interventions for autistic individuals using the serious game framework. *Research in Autism Spectrum Disorders, 66* doi:10.1016/j.rasd.2019.101412

Tavares, F. D. S., Azevedo, Y. J., Fernandes, L. D. M. M., Takeuti, A., Pereira, L. V., Ledesma, A. L. L., & Bahmad, F. (2021). Cochlear implant in patients with autistic spectrum disorder—a systematic review. *Brazilian Journal of Otorhinolaryngology, 87*(5), 601-619. doi:10.1016/j.bjorl.2020.11.020

Tekin-Iftar, E., Olcay, S., Sirin, N., Bilmez, H., Degirmenci, H. D., & Collins, B. C. (2021). Systematic review of safety skill interventions for individuals with autism spectrum disorder. *Journal of Special Education, 54*(4), 239-250. doi:10.1177/0022466920918247

Ung, D., Selles, R., Small, B. J., & Storch, E. A. (2015). A systematic review and meta-analysis of cognitive-behavioral therapy for anxiety in youth with high-functioning autism spectrum disorders. *Child Psychiatry and Human Development, 46*(4), 533-547. doi:10.1007/s10578-014-0494-y

Valencia, K., Rusu, C., Quiñones, D., & Jamet, E. (2019). The impact of technology on people with autism spectrum disorder: A systematic literature review. *Sensors (Switzerland), 19*(20) doi:10.3390/s19204485

Vanderkerken, L., Heyvaert, M., Maes, B., & Onghena, P. (2013). Psychosocial interventions for reducing vocal challenging behavior in persons with autistic disorder: A multilevel meta-analysis of single-case experiments. *Research in Developmental Disabilities, 34*(12), 4515-4533. doi:10.1016/j.ridd.2013.09.030

Walsh, C., O’Connor, P., Walsh, E., & Lydon, S. (2021). A systematic review of interventions to improve healthcare experiences and access in autism. *Review Journal of Autism and Developmental Disorders,* doi:10.1007/s40489-021-00279-2

Wang, D., Mason, R. A., Lory, C., Kim, S. Y., David, M., & Guo, X. (2020). Vocal stereotypy and autism spectrum disorder: A systematic review of interventions. *Research in Autism Spectrum Disorders, 78* doi:10.1016/j.rasd.2020.101647

Wang, X., Zhao, J., Huang, S., Chen, S., Zhou, T., Li, Q., . . . Hao, Y. (2021). Cognitive behavioral therapy for autism spectrum disorders: A systematic review. *Pediatrics, 147*(5) doi:10.1542/peds.2020-049880

Watkins, L., Ledbetter-Cho, K., O'Reilly, M., Barnard-Brak, L., & Garcia-Grau, P. (2019). Interventions for students with autism in inclusive settings: A best-evidence synthesis and meta-analysis. *Psychological Bulletin, 145*(5), 490-507. doi:10.1037/bul0000190

Williams, J. H. G., Whiten, A., & Singh, T. (2004). A systematic review of action imitation in autistic spectrum disorder. *Journal of Autism and Developmental Disorders, 34*(3), 285-299. doi:10.1023/B:JADD.0000029551.56735.3a

Williams, K., Wheeler, D. M., Silove, N., & Hazell, P. (2010). Selective serotonin reuptake inhibitors (SSRIs) for autism spectrum disorders (ASD). *The Cochrane Database of Systematic Reviews,* (8), CD004677. doi:10.1002/14651858.CD004677.pub2

Wolstencroft, J., Robinson, L., Srinivasan, R., Kerry, E., Mandy, W., & Skuse, D. (2018). A systematic review of group social skills interventions, and meta-analysis of outcomes, for children with high functioning ASD. *Journal of Autism and Developmental Disorders, 48*(7), 2293-2307. doi:10.1007/s10803-018-3485-1

Yang, D., Robertson, H. L., Condliffe, E. G., Carter, M. T., Dewan, T., & Gnanakumar, V. (2021). Rehabilitation therapies in Rett syndrome across the lifespan: A scoping review of human and animal studies. *Journal of Pediatric Rehabilitation Medicine, 14*(1), 69-95. doi:10.3233/PRM-200683
