# Supplementary material for: Mental health in autistic adults: A rapid review of prevalence of psychiatric disorders and umbrella review of the effectiveness of interventions within a neurodiversity informed perspective
Source: PLoS One. 2023 Jul 13;18(7):e0288275. doi: 10.1371/journal.pone.0288275 (PMC10343158; doi:10.1371/journal.pone.0288275)
Supplement: S3 File — (DOCX) [file pone.0288275.s004.docx]

**Outcome Measure- Full Name and Author, where reported:**-Corsi-BTT= Corsi Block Tapping Task, BRIEF= Behavioural Rating Inventory of Executive Functioning, SSRT= The stop-signal reaction time, CPT= Continuous Performance Test, WCST-Wisconsin Card Sorting Test (Heaton 1981), ScoRS-J=Schizophrenia Cognition Rating Scale – Japanese Version, MCCB=MATRICS Consensus Cognitive Battery, MSCEIT= The Mayer-Salovey-Carusco Intelligence Test (Meyer at al. 2002), PERT=Penn Emotion Recognition Test, PEDT= Penn Emotion Discrimination Task, PEAT = Penn Emotional Acuity Task, SCS= Social Cognition Profile, AQ= Autism Questionnaire, ASQ= Autism Spectrum Quotient, CAMM= Children Acceptance and Mindfulness Measure, CERQ=Cognitive Emotion Regulation Questionnaire, CSQ-CA= Chronic Stress Questionnaire for children and adolescents, CSRQ= Chronic Sleep Reduction Questionnaire, DASS/DASS 21= Depression Anxiety Stress Scale (Lovibond & Lovibond 1995), DERS= Difficulties in Emotional Regulation Scale, ESS= Somatic Symptom Scale, FFMQ= Five Facet Mindfulness Questionnaire, FMI= Freiburg Mindfulness Inventory, GHQ= General Health Questionnaire, GMS= Global Mood Scale, HADS= Hospital Anxiety and Depression Scale (Zigmond & Snaith, 1983), IBI= Irrational Beliefs Scale, IM-P= Interpersonal Mindfulness in Parenting Scale, MAAS= Mindful Attention and Awareness Scale, OQ= Outcome Questionnaire, POMS= Profile of Mood States, PS= Parenting Scale, PSI=Parenting Stress Index, PSS= Parenting Stress Scale, PSS= Perceived Stress Scale, PSWQ= Penn State Worry Questionnaire, RRQ=Rumination Reflection Questionnaire, RRS= Ruminative Response Scale, SCS= Self Compassion Scale, SCL-90/-R= Symptom Checklist 90/- Revised (Derogatis & Cleary, 1977), SRS= Social Responsiveness Scale, STAEI-2= State Trait Anger Expression Inventory, STAI-S= State Trait Anxiety Inventory, WHO-5= World Health Organisation Five Well Being Index, WHOQOL-BRIEF= World Health Organisation Quality of Life Assessment, ZBI= Zarit Burden Inventory, ABC= Aberrant Behavior Checklist, CGI-I/S= Clinical Global Impression Scale-Improvement/ Severity (Guy 1976), PANSS= Positive and Negative Symptom Scale, ADOS/ G= Autism Diagnosis Observation Schedule (Lord et al. 2000), FEAS= Functional Emotional Assessment Scale, ADI-R= Autism Diagnosis Interview- Revised (Rutter et al. 2003), ESCS- Early Social Communication Scales, IPR= Index of Peer Relations, ECA-R= Revised Clinical Scale for the Evaluation of Autistic Behaviour, PDDBI= PDD Behaviour Inventory, ATEC= Autism Treatment Evaluation Checklist, PPVT-4= Peabody Picture Vocabulary Test Fourth Edition, TEA-Ch= Test of Everyday Attention for Children, CCC-2= Children’s Communication Checklist- 2^nd^ Edition, CARS2-HF= High Functioning Version Rating Booklet, STAI= State Trait Anxiety Inventory (Spielberger et al. 1983), CDI-2= Children’s Depression Inventory, CCAPS-34= Counselling Center Assessment of Psychological Symptoms-34, RCADS= Revised Children’s Anxiety and Depression Scale, BASC-2= Behavioural Assessment System for Children, second Edition, CDI= Children’s Depression Inventory, HAM-D= Hamilton Depression Rating Scale, HAM-A= Hamilton Rating Scale for Anxiety, CDRS= Children’s Depression Rating Scale, MADRS= Montgomery-Asberg Depression Rating Scale, GPA=Grade Point Average, BRIEF-A= Behaviour Rating Inventory of Executive Function for Adults, D-KEFS= Delis-Kaplan Executive Function System, SSPA= Social Skills Performance Assessment, SRS-2= Social Responsiveness Scale-2, SFQ= Social Functioning Questionnaire, GSE= General Self-Efficacy Scale (Narita et al. 1995), PESE= Perceived Empathetic Self-Efficacy Scale, PHQ-9= Patient Health Questionnaire, GAD/GAD-7= Generalised Anxiety Disorder Questionnaire (Spitzer et al. 2006), SSRS= Social Skills Rating System (Gresham & Elliott 1990), SELSA= Social and Emotional Loneliness Scale for Adults (DiTommasso & Spinner 1993), EQ= Empathy Quotient, QSQ= Quality of Socializing Questionnaire (Frankel & Mintz 2008), SSI= Social Skills Inventory, TYASSK= Test of Young Adult Social Skills Knowledge (Laugeson & Frankel, 2010), RMET= Reading the Mind in the Eyes Test (Baron-Cohen et al. 1997; Baron-Cohen et al. 2001), ERG= Emotion Regulation Questionnaire, BPAQ= Buss and Perry Aggression Questionnaire, SPAI-23= Social Phobia and Anxiety Inventory-23, AQ= Autism Quotient, ASES= Academic Self-Efficacy Survey, DIOS= Disability Identity and Opportunities Scale, SRS-A= Social Responsiveness Scale- Adult, TONI-3= Test of Non-verbal Intelligence- Third Edition, ACS-SP= Advanced Clinical Solutions- Social Perception Subtest, ER40- Emotional Recognition 40, HCAS= Home and Community Activities Scale, SSIS-RS= Social Skills Improvement System – Rating Scales (Gresham & Elliott 2008), LSAS/ LSAS-SR= Liebowitz Social Anxiety Scale- Self Report (Liebowitz 1987), SPIN= Social Phobia Inventory, ABAS-3= Adaptive Behaviour Assessment System – 3^rd^ Edition, CSES= Coping Self-Efficacy Measure, SD= Self-Determination, ASEBA-ASR= ASEBA Adult Self-Report (Achenbach & Rescorla, 2003), URP-ASSET= User Rating Profile, SCI= Social Communication and Interaction, FEIT= The Face Emotion Identification Test, SCSQ= Social Communication Skills Questionnaire. PTQ= Parent Teacher Questionnaire, SOME= Summation of Maladaptive Expression, BDC= Berkson and Davenport Stereotypies Checklist, SSCQ= Social Skills Communication Questionnaire, BDI/ BDI-II= Beck Depression Inventory (Beck et al. 1996), BAI= Beck Anxiety Inventory (Beck et al. 1988), SPAI= Social Phobia and Anxiety Inventory, WSAS= Work and Social Adjustment Scale, YBOCS/ DYBOCS= Yale-Brown Obsessive Compulsive Scale (Goodman et al. 1989), SCID-IV= Structured Clinical Interview for Disorders, RCMAS= Revised Children’s Manifest Anxiety Scale, FSSC= Fear Survey Schedule for Children, VBAS/ VABS/ VABS-II Vineland Adaptive Behaviour Scales/ II (Sparrow et al. 1984), BASC 2PRS= Behaviour Assessment System for Children – 2 Parent Rating Scales, SPWSS= Social Phobia Weekly Summary Scale, RSE/RSES= Rosenberg Self-esteem Scale (Rosenberg 1965), CORE OM= Clinical Outcomes in Routine Evaluation, BSI= Brief Symptom Inventory, ASR= Adult Self Report, UCLAS= UCLA Loneliness Scale, IGIRT= Inference Generation in Reading Test (Murza 2011), TASIT/TA-SIT= The Awareness of Social Inference Test (McDonald et al. 2002), MIRI= Metacognition in Reading Inventory (Ehren 2008), GRADE= Group Reading Assessment and Diagnostic Evaluation (Williams 2001), ABAS3= Adaptive Behaviour Assessment System – Third edition (Harrison & Oakland 2015), SDSS= Self-Determination Skills Survey, BTFR= Benton Test of Facial Recognition (Benton et al. 1983), WMS= Wechsler Memory Scale, ERP= Event Related Potentials, STAI-T= State Trait Anxiety Inventory (Spielberger et al. 1983), CORE-OM= Outcomes in Routine Evaluation -Outcome Measure (Evans et al. 2000), QOL= Quality of Life Questionnaire (Caballo et al. 2005), DEX= Dysexecutive Questionnaire (Burgess et al. 1998), CANTAB= Cambridge Neuropsychological Test Automated Battery (Robbins et al. 1994), CHART= Craig Handicap Assessment and Rating Technique (Whiteneck et al 1992), Osnabruck Ability to Work Profile (Self-Report, Supervisors Report) (Wiedl & Uhlhorn, 2006), CSSCEI= Cognitive Style & Social Cognition Eligibility Interview (Hogarty et al. 2004), SIS= Supports Intensity Scale- Employment Subscale (Thompson et al. 2004), EPER= Employee Performance Evaluation Report (Van Bourgondien et al. 1998), SOC= Sense of Coherence Scale, MRI= Magnetic Resource Imaging, OCI-R= Obsessive Compulsive Inventory- Revised (Foa et al. 2002), WSAS= Work and Social Adjustment Scale, ERSES= Emotion Recognition Self-efficacy Scale, ERS= Environment Rating Scale (Van Bourgondien et al. 1998), AAPEP= Adult Psychoeducational Profile, ABI= Autism Behaviour Inventory, BDEFS= Barkley Deficits in Executive Functioning Scale (Barkley 2012), SACQ= Student Adaptation to College Questionnaire (Van Bourgondien et al. 1998), LASMI= Life Assessment Scale for the Mentally Ill (Iwasaki et al. 1994), CHOCHI-R= Obsessive Compulsive Inventory- Parent (Shafran et al. 2003), FAS-PR= Family Accommodation Scale-Parent Report (Storch et al. 2007), SCQ= Social Communication Questionnaire, SDS= Self-Determination Questionnaire, ASRS= Adult ADHD Self-Report Scale (Kessler et al. 2005), CPT= Continuous Performance Test (Rosvold et al. 1965), MASC= Movie for the Assessment of Social Cognition (Dziobek et al. 2006), MESSIER= Matson Evaluation of Social Skills for Persons with Severe Retardation (Matson, 1995), SIS= Supports Intensity Scale, MRAI= Major Role Adjustment Inventory (Hogarty et al. 1974), BAFT= Believability of Anxious Feelings and Thoughts Questionnaire (Herzberg et al. 2012), BACS-J= Brief Assessment of Cognition in Schizophrenia- Japanese Version (Kaneda et al. 2007), CSES= Coping Self-Efficacy Scale (Chesney et al. 2006), DGMS/ GMS= Dutch Global Mood Scale (Denollet 1993), CFQ= Cognitive Fusion Questionnaire (Gillanders et al. 2014), SCAS= Spence Children’s Anxiety Scale (Spence 1998), IRI= Interpersonal Reactivity Index (Davis 1983), SSS= Stress Survey Schedule for Persons with Autism and Other Pervasive Developmental Disabilities (Groden et al. 2001), PWI-ID= Personal Well-being Index (Cummins et al 2010), CEEQ= Cognitive and Emotional Empathy Questionnaire (Savage et al, no date), ASD-DA= Autism Spectrum Disorders – Diagnosis for Adults (Matson et al. 2008), QOLI= Quality of Life Inventory (Frisch 1994), QoL-Q- Abridged= Quality of Life Questionnaire -Abridged (Shalock & Keith 1993), SPS= Social Pragmatic Scale (Morgan 2011), GAF= Global Assessment of Functioning (Piersma & Boes, 1997), CY-BOCS= Children’s Yale Brown Obsessive Compulsive Scale (McKay 2003), Y-BOCS= Yale- Brown Obsessive Compulsive Scale (Goodman 1989), CY-BOCS-PDD= Children’s Yale Brown Obsessive Compulsive Scale modified for children with pervasive developmental disorders (Scahill 2006), SRS= Social Responsiveness Scale (Constantino & Gruber 2005), WFIRS-S= Weiss Functional Impairment Rating Scale – Self-Report, SRS=Social Responsiveness Scale, PESE= Perceived Empathy self-efficacy, PSSE= Perceived Social Self-efficacy, SCSQ= Social Communication Skills Questionnaire, SSPA= Social Skills Performance Assessment, MSCS-C= Multidimensional Social Competence Scale- Chinese version, CCAPS= Assessment of Psychological Symptoms, SRS=Social Responsiveness Scale (Constantino& Gruber 2005).
