# Supplementary material for: Mental health in autistic adults: A rapid review of prevalence of psychiatric disorders and umbrella review of the effectiveness of interventions within a neurodiversity informed perspective
Source: PLoS One. 2023 Jul 13;18(7):e0288275. doi: 10.1371/journal.pone.0288275 (PMC10343158; doi:10.1371/journal.pone.0288275)
Supplement: Attachment — Submitted filename: Response to Reviewers.docx [file pone.0288275.s006.docx]

**PONE-D-22-2007**

Dear Editor

**Mental health in autistic adults: a rapid review of prevalence of psychiatric disorders and umbrella review of the effectiveness of interventions within a neurodiversity informed perspective**

Thank you for considering this manuscript for publication in PLOS ONE.

Following review, we have made extensive changes to this manuscript including updating searches to November 2022 for both reviews. We have considered all the points made by the reviewers and have altered the manuscript accordingly.

We would like to thank the reviewers for the consideration they have given to our manuscript and for sharing their extensive expertise to enable us to improve the quality of this document.

In the attached table we have described the amendments we have made to this manuscript in response to points raised by the reviewers together with the location where they occur.

We look forward to hearing from you in due course.

Yours faithfully

Eleanor Curnow

Research Fellow, Queen Margaret University, Edinburgh.

| **Reviewer Remark** | **Author** | **Manuscript Amendment** | **Location** |
| --- | --- | --- | --- |
| Abstract 1. Explaining why understanding mental health problems is important (e.g., to inform funding of supports, to support better quality of life) in background would be useful | We have added detail regarding the importance of mental health problems to the manuscript. | Autistic adults have high risk of mental ill-health and some available interventions have been associated with increased psychiatric diagnoses. Understanding prevalence of psychiatric diagnoses is important to inform the development of individualised treatment and support for autistic adults which have been identified as a research priority by the autistic community. Interventions require to be evaluated both in terms of effectiveness and regarding their acceptability to the autistic community. | Page 2, lines 27-32 |
| Introduction 2. The introduction is quite limited and further information to set the context for the study and the lens through which it was written would be valuable. Suggested additions are: 1. Providing a definition of mental health vs. psychiatric conditions and explaining the selection of conditions included in the review. Then using the same term throughout as I note use of both terms. | We have enhanced the introduction as advised by the reviewer. | Mental health is a state of well-being in which an individual realises his or her own abilities, can cope with the normal stresses of life, can work productively and is able to make a contribution to his or her community [15].  For the purposes of this review a psychiatric disorder is defined as a mental illness diagnosed by a mental health professional according to diagnostic criteria [17]. Relevant diagnoses were identified according to search terms and strategies described by Cochrane Common Mental Disorders [18]. | P4, 94-97,  P5, 102-105 |
| 2. Overviewing prevalence of the above included conditions for neurotypical populations to provide a context for comparisons of prevalence | We have provided additional detail on prevalence as advised by the reviewer. | Worldwide prevalence of psychiatric disorders is estimated at 13%, including anxiety disorders (4.1%), depressive disorders (3.8%), bipolar disorders (0.5%), schizophrenia (0.3%), and eating disorders (0.2%) [10] . In Scotland, census data indicates that 5.4% of adults aged 16-64 years (4.6% for people aged 65+) without co-occurring intellectual disabilities and autism reported mental ill-health which had lasted or was expected to last at least 12 months [8]. | P4, 82-86 |
| 3. Defining the neurodiversity paradigm as well as previous research on priorities of the autistic community for research and previous research into preferred outcomes for treatments of relevance in interpreting results. | We have added information on the neurodiversity paradigm and priorities of the autistic community. | The ‘neurodiversity’ movement considers autism and other neurodevelopmental conditions as neurological variation, rather than disorders requiring treatment [2, 23, 24], Therefore, autism is a difference not a deficit, which brings into question the use of interventions which seek to ‘cure, fix or normalise’ [2]. This movement has provided tools to critique research and to consider what is important in research and practice for autistic adults [16, 24, 25]. This has led to the development of research priorities which focus on the best interests of autistic people, and recognise that the inclusion of both autistic people and non-autistic people in research processes is of key importance [23]. Although, there is a need for progress as only 5% of funded autism research included autistic adults [26]. Historical research must be reviewed through a contemporary lens which considers the acceptability of terminology, interventions, supports and outcomes to the autistic community [24]. Research indicates that autistic people prioritise outcomes associated with quality of life, reduction in anxiety, depression or sleep related problems, social well-being, interpersonal relationships, and increased participation in activities of daily living, community and work [25]. | P5, 117-130 |
| 4. Defining evidence-based practice including differentiating classification based on evidence/GRADE criteria as used in the methods vs. the broader EBP framework incorporating the integration of research evidence, client priorities and preferences and practitioner expertise (cf. Sackett for example). | We have added further definition of evidence based practice to the manuscript. | These measures are key to evidence-based practice which requires the integration of the best available research with clinical expertise and the patient’s unique values and circumstances [27, 28]. Evidence based practice requires that health care is not only based upon the best available, valid and current evidence as defined by GRADE, but also that decisions are made by those receiving care and informed by those providing care [28, 29]. | P5, 131-135 |
| 5. An overview of previous similar reviews in children, e.g., umbrella review of interventions e.g., i. Trembath, D., Varcin, K., Waddington, H., Sulek, R., Bent, C., Ashburner, J., ... & Whitehouse, A. (2022). Non-pharmacological interventions for autistic children: An umbrella review. Autism, 13623613221119368. ii. Hossain, M. M., Khan, N., Sultana, A., Ma, P., McKyer, E. L. J., Ahmed, H. U., & Purohit, N. (2020). Prevalence of comorbid psychiatric disorders among people with autism spectrum disorder: An umbrella review of systematic reviews and meta-analyses. Psychiatry Research, 287, 112922. | We have detailed previous umbrella reviews within the manuscript. | i. A recent umbrella review found that research evidence did not support one best intervention for autism in children, and that there was a concerning lack of consideration of adverse effects of interventions [20].  ii. Whilst there has been previous consideration of prevalence of psychiatric disorders in autistic populations [11], there is a need to distinguish between adult and child populations. | P5, 112-114,  P4, 87-88 |
| 6. I would like to see discussion of how this review is needed given 5.ii and what this adds (e.g., the review of interventions/autistic input?) | 5.ii refers to an umbrella review of prevalence which does not differentiate between adults and children.  We have also enhanced the argument for further review of interventions for autistic adults. | Whilst there has been previous consideration of prevalence of psychiatric disorders in autistic populations [11], there is a need to distinguish between adult and child populations.  There is limited understanding of effective interventions for supporting mental health in autistic adults [2]. A recent umbrella review found that research evidence did not support one best intervention for autism in children, and that there was a concerning lack of consideration of adverse effects of interventions [20]. Previous research has focussed on children and adolescents, often evaluating interventions designed to reduce or mask behaviours associated with autism [20] but there is now recognition of the stress and detriment such interventions can create [21, 22]. The ‘neurodiversity’ movement considers autism and other neurodevelopmental conditions as neurological variation, rather than disorders requiring treatment [2, 23, 24], Therefore, autism is a difference not a deficit, which brings into question the use of interventions which seek to ‘cure, fix or normalise’ [2]. This movement has provided tools to critique research and to consider what is important in research and practice for autistic adults [16, 24, 25]. This has led to the development of research priorities which focus on the best interests of autistic people and recognise that the inclusion of both autistic people and non-autistic people in research processes is of key importance [23]. Although, there is a need for progress as only 5% of funded autism research included autistic adults [26]. Historical research must be reviewed through a contemporary lens which considers the acceptability of terminology, interventions, supports and outcomes to the autistic community [24]. Research indicates that autistic people prioritise outcomes associated with quality of life, reduction in anxiety, depression or sleep related problems, social well-being, interpersonal relationships, and increased participation in activities of daily living, community, and work [25]. | P4, 87-88,  P5, 111-130 |
| 3. Please define and justify the use of a rapid review? | We have added details justifying the use of rapid review to the manuscript. | The prevalence of psychiatric disorders in autistic adults will be explored through rapid review of published literature. A rapid review has been defined as “a rigorous and transparent form of knowledge synthesis which accelerates the process of conducting a traditional systematic review through streamlining or omitting a variety of methods to produce evidence for stakeholders in a resource-efficient manner” [19]. | P5, 106-110 |
| 4. Alternatively, I wonder if the review of interventions may be more appropriately described as an umbrella review (see below) rather than a rapid review? See e.g., 1. Aromataris, E., Fernandez, R., Godfrey, C. M., Holly, C., Khalil, H., & Tungpunkom, P. (2015). Summarizing systematic reviews: methodological development, conduct and reporting of an umbrella review approach. JBI Evidence Implementation, 13(3), 132-140.  The inclusion of previous systematic reviews in your review was interesting - what reason drove this decision? typically previous reviews are excluded unless it is specifically a review or reviews. I wonder if focusing purely on previous reviews, elements of individual studies examining interventions were missed. I assume the rationale is that there would be too many studies, and this would then lead to the question as to whether this paper could be two separate reviews? I am not necessarily saying that this needs to be the case but clearer articulation of decision making would help here. | The review of systematic reviews of interventions has now been described as an umbrella review throughout the manuscript. Additionally umbrella review has been described within the manuscript: | This umbrella review of interventions will therefore consider the results of studies not only in terms of their effectiveness, but also regarding the acceptability of the interventions to the autistic community [23].  An umbrella review facilitates a synthesis and appraisal of evidence across a broader topic area than can usually be achieved through an individual systematic review [29]. | P5/6 135-138 |
| 5. Please provide references for assertions on page 4-5 lines 86-87 and 88-98 . | Additional references have been added. |  | P5 117-130 |
| 6. I would be interested to see research questions as well as aims. | We have added research questions as requested by the reviewer. | Research Questions:  1. How prevalent are psychiatric diagnoses in autistic adults?  2. Which factors are associated with heterogeneity of prevalence of psychiatric diagnoses in autistic adults?  3. Which interventions are effective in treating autistic adults?  4. Do available interventions meet the needs and priorities of autistic adults? | P6 157-162 |
| Method 7. I am concerned at the relatively small number of articles found in searches, please discuss/justify selection of databases and whether likely to have captured all available studies? | Additional description regarding the selection of databases has been added. | Databases were selected from resources available at Queen Margaret University which were known to publish most systematic reviews following discussion with university research librarian and with reference to published guidance [33, 34]. | P7 190-192 |
| 8. I understand that this is a rapid review but I am left wondering what the decision making was behind the lower date range of 2011? Is there a significant event at this time that makes papers prior to this redundant? I think some justification needs to be provided for this decision. | Citation supporting this decision has been added. | Search date was restricted to 10 years as this is a valid and reliable approach for rapid reviews [34]. | P7, 170-171 |
| 9. I note the searches were conducted in November 2021, given use of systematic reviews that likely completed searches 6-12 months prior to their publication I am concerned about the recency of information, as such I suggest updating the searches and manuscript. | Searches have been updated to November 2022. This is reflected in the manuscript and results tables. |  |  |
| 10. Extraction of ability level (e.g., verbal, adaptive functioning, intellectual ability) in studies is recommended where available to determine representativeness of samples | Prevalence of Intellectual Disability has been added to Prevalence study Characteristics.  Ability level, where reported, has been added to systematic reviews of interventions table. |  | Table 2  Table 3 |
| 11. Blind coding of risk of bias and reporting of Cohen’s Kappa is recommended | There was blind coding of risk of bias and this has now been made clear in the manuscript. Inter-rater agreement was assessed using Cohens Kappa. |  | P7, 196 and 199-200 |
| 12. It is unclear the process by which autistic input on intervention outcomes/suitability in regard to the neurodiversity paradigm (p. 8) was completed, please describe, including inter-rater reliability/confirmation of consistency of ratings/determination of not recommended. | We have provided additional description regarding the inclusion of autistic researchers in this study. | Autistic researchers were included throughout the rapid review and umbrella review process and provided critique of evidence for interventions which could potentially be detrimental to autistic people, including encouraging the expression of neurotypical behaviours which is a form of masking. There were no incidences of disagreement between research team members regarding the classification of evidence for interventions. Papers were examined for the inclusion of autistic researchers, and for views expressed by autistic people on the acceptability of interventions. | P41, 501-507 |
| Results 13. For prevalence studies, where reported it would be useful to include further descriptors of participants such as whether findings differed by presence of co-occurring intellectual disability and noting if this was not described. | Percentage of population with Intellectual Disability, where reported, has been added to Prevalence Study Characteristics Table. |  | Table 2 |
| 14. There is limited information provided in table 3 and reporting of classification of interventions to determine GRADE criteria- I am interested in the number of RCTs in each paper, the outcome measure/s, mental health conditions, and whether studies found positive, negative, or mixed results. At present there is not enough information to verify the classifications (e.g., that no intervention meets criteria as evidence-based) | Additional information (where reported) regarding ability level, outcome measures, and condition targeted has been added to table describing the characteristics of systematic reviews of interventions for autistic adults. Number of RCT’s is reported in included studies column. |  | Table 2 |
| 15. Page 26, line 289 noted “none of the community council of autistic researchers felt this was an appropriate intervention…” as per request in method above, outlining how this process was completed and documented is needed, description of findings for each intervention would also be valuable. | We have added additional information regarding the included study which employed the community council of autistic researchers. | Exceptionally, one paper described the inclusion of autistic researchers within the research process [59]. This study included a community council comprising 18 people who mostly identified as autistic or were the parent of an autistic adult, and were researchers, medical or mental health professionals, authors, or advocates. This council reviewed study results and contributed to study recommendations [59]. | P21, 276-280 |
| Discussion 16. Gender imbalance (line 297-298) should be outlined in the introduction and reference previous research. | We have added information regarding gender imbalance to the introduction. | Estimated prevalence of autism in adults aged 16-64 years in UK is 2.9% [95% CI 2.7, 3.1] [4]. Prevalence of autism is 3.46 times higher for boys [5]. | P4, 74-75 |
| 17. Additional review in introduction is needed to then compare observed prevalence to neurotypical population (e.g., line 299-300 to discuss if these rates are higher/lower) | We have described overall prevalence of psychiatric disorders in the introduction. | Worldwide prevalence of psychiatric disorders is estimated at 13%, including anxiety disorders (4.1%), depressive disorders (3.8%), bipolar disorders (0.5%), schizophrenia (0.3%), and eating disorders (0.2%) [10] . In Scotland, census data indicates that 5.4% of adults aged 16-64 years (4.6% for people aged 65+) without co-occurring intellectual disabilities and autism reported mental ill-health which had lasted or was expected to last at least 12 months [8]. | P4, 82-86 |
| 18. Age-related differences in lifetime prevalence likely relate to more opportunities to experience challenges with increasing age, this possibility is not discussed (see lines 309-312) | This possibility is now indicated in the manuscript. | Autistic people over the age of 65 were more likely to report a lifetime mental health condition than autistic people aged 55-65, although this pattern was not found when examining current diagnoses [52] possibly due to additional time available to experience mental ill-health. | P38/39, 425-428 |
| 19. Discussion of elevated rates of mental health challenges in autistic people with intellectual disability vs. without should have been overviewed in the introduction (lines 313-315). | Needs of autistic people with intellectual disability are now overviewed in the introduction. | Autistic people have a wide range of needs which vary depending on environment, and co-occurrence of intellectual or physical factors, sensory factors, co-occurring neurodevelopmental differences, intellectual disabilities, or other psychiatric diagnoses [6-8]. Autistic people, and people with intellectual disabilities have more mental and physical needs than other people [9], and research indicates that needs prevalence will be even higher for people with co-occurring autism and intellectual disability [8]. | P4, 76-81 |
| 20. Discussion of limitations of current measures to detect mental health difficulties in this population (lines 316-320) would also be useful to discuss to set the scene in the introduction; overview of included measures would also be useful in the results to then critically evaluate if previous research has shown validity in this population to critically evaluate the previous findings. | Additional discussion of outcome measures has been added to the introduction. The limited description of the measures prevented further investigation. | Further consideration of the measurement tools used with autistic adults is also required to ensure that they are validated for this population [12].  Outcome measures were often not referenced adequately to permit investigation into their reliability or validity for this population. | P4, 88-90  P21, 276-280 |
| 21. Lines 321-313 discuss the lack of inclusion of autistic individuals in research- this is not clearly outlined in the results and should be added there and how this was determined (e.g., review of paper authorship, methods). It should also be acknowledged that research teams may include autistic individuals who chose not to self-disclose, unless it is explicitly stated that autistic researchers were not involved with community involvement statements only recently mandated and only in select journals (e.g., Autism). | We have added further description on the inclusion of autistic individuals and a statement regarding the absence of a statement regarding the inclusion of autistic researchers in included studies. | Papers were examined for the inclusion of autistic researchers, and for views expressed by autistic people on the acceptability of interventions.  Retrieved studies did not declare that they did not include autistic researchers and research teams may therefore have included autistic researchers. | P41, 505-507, and 514-516 |
| 22. The range of outcomes and heterogenous tools is outlined on line 338- these should be outlined in the results (e.g. outcomes and tools/measures added to Table 2) | Outcomes and tools/ measures have been added to intervention study characteristics table. |  | Table 2 |
| 23. Implications are not linked to previous research/findings- line 355-374 needs to link for example to autistic priorities (e.g., priority setting studies) and/or previous research findings. | Additional references have been added linking implications to previously published research/ findings. |  | P42, 474-499 |
| 24. Line 385-386 notes “other reviewers could come to different conclusions.” To address this limitation, further information about how conclusions were drawn so findings could be replicated in the method is needed, otherwise this is a serious limitation. | Additional information regarding the application of GRADE criteria has been provided. | Interventions were evaluated against the stated adapted GRADE criteria to determine not only evidence of effectiveness but also evidence of negative consequences or harm. Where either of these processes indicated concern regarding the acceptability of interventions, they were categorised as not recommended. Evidence which contradicted current clinical guidelines or has been classified as not acceptable to the autistic community was not recommended. | P9, 236-239 |
