# Supplementary material for: Mental health in autistic adults: A rapid review of prevalence of psychiatric disorders and umbrella review of the effectiveness of interventions within a neurodiversity informed perspective
Source: PLoS One. 2023 Jul 13;18(7):e0288275. doi: 10.1371/journal.pone.0288275 (PMC10343158; doi:10.1371/journal.pone.0288275)
Supplement: Attachment — Submitted filename: Response to Reviewer (2).docx [file pone.0288275.s007.docx]

Response to Reviewers

The authors wish to thank the reviewers for their in-depth consideration of this manuscript. All points raised by reviewers are addressed below including details of manuscript amendments.

Introduction
1. The definition of a rapid review does not quite fit where it is placed in text (p. 5, line 105-109), I suggest moving it to p. 6 with discussion of the umbrella review component, paraphrasing instead of directly quoting, and operationalising what about this review specifically made it a rapid review as opposed to a systematic review. Some of this information is included later in the methods but I felt this would be valuable together to set the scene for the paper.

The authors have moved the statement defining rapid review as advised by reviewer:

P5, line 127-131 The prevalence of psychiatric disorders in autistic adults will be explored through rapid review of published literature. This knowledge synthesis will be rigorous and transparent but will be accelerated through the use of resource-efficient methods including limiting the number of databases which will be searched for evidence. Handsearching, and forward and backward citation searches will also not be undertaken[30].

2. GRADE acronym is not defined or operationalised on p. 5 which I suggest unpacking.

The authors have amended the text as follows:

P5, line 120-123 Evidence based practice requires that health care is not only based upon the best available, valid, and current evidence as defined by GRADE[28] (Grading of Recommendations, Assessment, Development and Evaluation)[28], but also that decisions are made by those receiving care and informed by those providing care[27, 29].

Method
3. I continue to be concerned at the relatively small number of articles found in searches, please discuss/justify selection of databases and/or highlight this more strongly as a limitation in the discussion.

The authors have highlighted limitations in databases used within the limitations of the study:

P51, line 522-528 Rapid review methodologies were used[33]; including date and language restrictions, limiting the number of databases that were searched, and focussing on systematic reviews of intervention studies. The research team did not have access to EMBASE which is recommended for intervention reviews where available to researchers [38]. While the review team completed a comprehensive search using recognised methods no forward or backward citation search, hand searching or follow up with authors was completed to identify missing studies. These methods may have reduced the number of studies identified.

4. P. 9 it is noted that “not only evidence of effectiveness but also evidence of negative consequences or harm.” Table 1 outlines how evidence of effectiveness was assessed but how was evidence of negative consequences or harm evaluated?

The authors have provided additional information regarding the evaluation of negative consequences or harm:

P9, line 229-240

Interventions were evaluated against the stated adapted GRADE criteria to determine not only evidence of effectiveness, but also evidence of negative consequences or harm. This involved consideration of reported benefit and adverse effects for each intervention type. Interventions which focussed on the reduction of core features of autism are associated with harmful consequences and contradict current clinical guidelines so were rated as not recommended[42]. All members of the research team which included autistic researchers, were involved in this process. Arising disagreements were resolved through team discussion with reference to the principles of the neurodiversity paradigm[43] and expressed priorities and concerns of autistic adults[14, 44, 45]. Where either of these processes indicated concern regarding the acceptability of an intervention, it was categorised as not recommended. Interventions which contradicted current clinical guidelines were also not recommended.

5. It remains unclear the process by which autistic input on intervention outcomes/suitability in regard to the neurodiversity paradigm (p. 9) was completed, please describe, including inter-rater reliability/confirmation of consistency of ratings/determination of not recommended.

- The authors have enhanced the description of negative consequences or harm to clarify the connection between the neurodiversity paradigm, neurodiversity affirming practice and consequences of intervention (see response above).
- There is no calculation of inter-rater reliability as this process was applied to interventions across different systematic review studies.
- Ratings were agreed by the entire team, following discussion as described on P9, line 234-237

All members of the research team which included autistic researchers, were involved in this process. Arising disagreements were resolved through team discussion with reference to the principles of the neurodiversity paradigm[43] and expressed priorities and concerns of autistic adults[14, 44, 45].

6. While Cohen’s Kappa (0.70) is acceptable it indicates there were a number of discrepancies, how were these handled?

P7, line 191-192

Disagreements were to be resolved through discussion and reference to a third party (MR) but this was not required.

Results
7. Table 3 has been expanded to provide additional information, however there remains limited information provided about the study findings (effectiveness: positive, negative and/or mixed results AND negative consequences- not reported) to determine GRADE criteria. At present there is not enough information to verify the classifications (e.g., that no intervention meets criteria as evidence-based).

P23, Table 3: Additional columns have been added to this table to show (1) Effectiveness (+, - or Mixed), and (2) Negative Consequences of Interventions.

8. I continue to be interested in the input and outcomes of the community council of autistic researchers for each intervention which based on the methods was used to inform GRADE classifications but does not seem to have been reported in the results? This would be valuable information to include.

The authors wish to state for clarification, a community council of autistic researchers is not included in methods of this study. The community of council of autistic researchers referred to in the text within the results section were part of one of the reported studies and not this review. However, the research team responsible for this study included autistic researchers. Table 4 includes the GRADE rating, together with key issues relating to the acceptability of interventions to autistic adults agreed following team discussion.

Discussion
9. Page 39, line 446-447, “A key issue was acceptability to the autistic community.” As per point 8 above these findings should be outlined in the results section.

Table 4 Evidence for Interventions

Column 3 has been extended to include additional information regarding the acceptability of interventions to autistic adults.

10. Similarly, p. 41, “Autistic researchers….provided critique of evidence for interventions which could potentially be detrimental to autistic people, including encouraging the expression of neurotypical behaviours…”- this critique should be outlined in the results section for each study (e.g., add to Table 3 and summarise findings across studies).

Table 4 Evidence for Interventions

Column 3 of this table has been amended to include information considered by the research team when making decisions regarding the acceptability of interventions to autistic adults.
