# Supplementary material for: Mental health in autistic adults: A rapid review of prevalence of psychiatric disorders and umbrella review of the effectiveness of interventions within a neurodiversity informed perspective
Source: PLoS One. 2023 Jul 13;18(7):e0288275. doi: 10.1371/journal.pone.0288275 (PMC10343158; doi:10.1371/journal.pone.0288275)
Supplement: Attachment — Submitted filename: Reviewer Rebuttal.docx [file pone.0288275.s008.docx]

Mental health in autistic adults: a rapid review of prevalence of psychiatric disorders and umbrella review of the effectiveness of interventions within a neurodiversity informed perspective

The authors wish to thank the reviewers and the editor for their consideration of this study. The authors have extensively revised the manuscript in response to their suggestions. Please find our responses to your suggested revisions below. We look forward to hearing your decision regarding publication of this manuscript in due course.

Journal Requirements:

**Please review your reference list to ensure that it is complete and correct. If you have cited papers that have been retracted, please include the rationale for doing so in the manuscript text, or remove these references and replace them with relevant current references. Any changes to the reference list should be mentioned in the rebuttal letter that accompanies your revised manuscript. If you need to cite a retracted article, indicate the article’s retracted status in the References list and also include a citation and full reference for the retraction notice.**

Author Response:

We have reviewed our reference list and applied citations using Endnote 20. This software has not indicated any retracted papers within our citation list.

Reviewers' comments:

Introduction

**1. The definition of a rapid review does not quite fit where it is placed in text (p. 5, line 105-109), I suggest moving it to p. 6 with discussion of the umbrella review component, paraphrasing instead of directly quoting, and operationalising what about this review specifically made it a rapid review as opposed to a systematic review. Some of this information is included later in the methods but I felt this would be valuable together to set the scene for the paper.**

Author Response:

P6, line128-134 We previously moved definition of rapid review to its current location prior to the description of the umbrella review as requested. We have added further details regarding the rapid review methods used within the study.

*The prevalence of psychiatric disorders in autistic adults will be explored through rapid review of published literature. This knowledge synthesis will be rigorous and transparent but will be accelerated by resource-efficient methods including limiting the number of databases which will be searched for evidence. Handsearching, and forward and backward citation searches will also not be undertaken[30]. Grey literature, and literature not published in English will not be considered. Article screening will be reviewed by two authors in 20% of publications.*

**2. GRADE acronym is not defined or operationalised on p. 5 which I suggest unpacking.**

Author Response:

P5, line 120-124

*Evidence based practice requires that health care is not only based upon the best available, valid, and current evidence as defined by GRADE (Grading of Recommendations, Assessment, Development and Evaluation)[28], but also that decisions are made by those receiving care and informed by those providing care[27, 29]. Strong GRADE evidence indicates all or almost all people would choose that intervention[30].*

Method

**3. I continue to be concerned at the relatively small number of articles found in searches, please discuss/justify selection of databases and/or highlight this more strongly as a limitation in the discussion.**

Author response:

P7, line 186-192

*Databases were selected from available resources following current guidance[37], and through discussion with the university research librarian. CENTRAL, MEDLINE and Embase (if access to Embase is available to the review team) are recommended for systematic reviews[37-39] . Embase was not available to the research team. Cochrane Database of Systematic Reviews was included as a major repository of systematic reviews[32]. Trials searches of JBI Database of Systematic Reviews and Implementation Reports did not reveal any additional relevant citations and was therefore excluded.*

P52, line 538-544 Additionally we have provided additional information in the limitations section highlighting the possible impact of rapid review methods including database limitations on the number of identified studies.

*Limitations*

*Rapid review methodologies were used[34]; including date and language restrictions, limiting the number of databases that were searched, and focussing on systematic reviews of intervention studies. The research team did not have access to EMBASE which is recommended for intervention reviews where available to researchers[37]. While the review team completed a comprehensive search using recognised methods no forward or backward citation search, hand searching or follow up with authors was completed to identify missing studies. These methods may have contributed to the low number of studies identified.*

**4. P. 9 it is noted that “not only evidence of effectiveness but also evidence of negative consequences or harm.” Table 1 outlines how evidence of effectiveness was assessed but how was evidence of negative consequences or harm evaluated?**

Author Response

P9, line 238-241 The authors have added additional information regarding their approach to the consideration of adverse outcomes.

*An exploratory approach was used to review adverse outcomes identified during the conduct of the review. This opportunistic approach considers only the reported adverse effects or outcomes that may be associated with the interventions being investigated[43].*

P52, line 550-554 The authors have also added the following limitations in this regard.

*An exploratory approach to reviewing adverse effects considers only reported information, and is therefore restricted by incomplete reporting, or inadequate monitoring of adverse outcomes. Separate searches for adverse effects of interventions were not conducted and therefore results are unlikely to be comprehensive[42].*

**5. It remains unclear the process by which autistic input on intervention outcomes/suitability in regard to the neurodiversity paradigm (p. 9) was completed, please describe, including inter-rater reliability/confirmation of consistency of ratings/determination of not recommended.**

Author Response

P9, line 241-249 We have simplified the description of this process and hope that it is now clearer to the reader.

*Interventions which focussed on the reduction of core features of autism are associated with harmful consequences and contradict current clinical guidelines so were rated as not recommended [44]. Core features include qualitative differences and impairments in reciprocal social interaction and social communication, restricted interests and activities, and rigid and repetitive behaviours[45]. Interventions which contradicted current clinical guidelines were also not recommended. All members of the research team which included autistic researchers, were involved in this process. Arising disagreements were resolved through team discussion. Inter-rater reliability was not recorded for this process.*

6**. While Cohen’s Kappa (0.70) is acceptable it indicates there were a number of discrepancies, how were these handled?**

Author Response

P8, Line 198-199

*Disagreements were resolved through discussion, and reference to a third party (MR) was not required.*

Results

7. **Table 3 has been expanded to provide additional information, however there remains limited information provided about the study findings (effectiveness: positive, negative and/or mixed results AND negative consequences- not reported) to determine GRADE criteria. At present there is not enough information to verify the classifications (e.g., that no intervention meets criteria as evidence-based).**

Author Response.

Negative consequences are reported in Table 3 Column 9. Additional information regarding the effect sizes of meta-analysis or quantitative synthesis conducted within the retrieved systematic reviews are reported in Table 3 Column 8.

Additionally we have included supplementary data describing overlap of primary studies included in the retrieved systematic reviews.

S3 File Primary Studies included in Systematic Reviews

8. **I continue to be interested in the input and outcomes of the community council of autistic researchers for each intervention which based on the methods was used to inform GRADE classifications but does not seem to have been reported in the results? This would be valuable information to include.**

Author response.

As stated in the previous rebuttal, this review did not include a community council of autistic researchers, although autistic researchers were integrated into the research team and included in all aspects of the review. Community council of autistic researchers is not included in the methods.

Discussion

9. **Page 39, line 446-447, “A key issue was acceptability to the autistic community.” As per point 8 above these findings should be outlined in the results section.**

Author Response.

P52, line 433-437

*One systematic review, exceptionally, reported including autistic individuals and families who checked results, recommendations, and acceptability of interventions[64]. Evidence for interventions aimed at the reduction of core features of autism were not recommended, Intervention studies were limited by not restricted reporting of outcome measures, or use of outcome measures not validated for autistic adults.*

**10. Similarly, p. 41, “Autistic researchers….provided critique of evidence for interventions which could potentially be detrimental to autistic people, including encouraging the expression of neurotypical behaviours…”- this critique should be outlined in the results section for each study (e.g., add to Table 3 and summarise findings across studies).**

Author Response

Table 4 Column 3 includes details regarding the acceptability of interventions to the autistic community. As most of the retrieved studies included a number of different types of interventions the authors have included this information in Table 4 which provided a summary of interventions.
