# Supplementary material for: Mental health in autistic adults: A rapid review of prevalence of psychiatric disorders and umbrella review of the effectiveness of interventions within a neurodiversity informed perspective
Source: PLoS One. 2023 Jul 13;18(7):e0288275. doi: 10.1371/journal.pone.0288275 (PMC10343158; doi:10.1371/journal.pone.0288275)
Supplement: Attachment — Submitted filename: Reviewer Rebuttal.docx [file pone.0288275.s009.docx]

Method

3**. I continue to be concerned at the relatively small number of articles found in searches, please discuss/justify selection of databases and/or highlight this more strongly as a limitation in the discussion.**

Author Response:

We have discussed the selection of databases (p7, line 186-191). We have provided additional reference to database selection as a concern within the limitations section of the manuscript.

P61, line 589-596

*Rapid review methodologies were used [34]; including date and language restrictions, limiting the number of databases that were searched, and focussing on systematic reviews of intervention studies.* *The research team did not have access to EMBASE which is recommended for intervention reviews where available to researchers [37]. While the review team completed a comprehensive search using recognised methods no forward or backward citation search, hand searching or follow up with authors was completed to identify missing studies.* *These methods and the selection of databases used in the search may have contributed to the low number of studies identified.*

5. **It remains unclear the process by which autistic input on intervention outcomes/suitability in regard to the neurodiversity paradigm (p. 9) was completed, please describe, including inter-rater reliability/confirmation of consistency of ratings/determination of not recommended.**

Author Response:

We have provided additional detail regarding the principles by which we considered the acceptability of interventions, in addition to criteria included in Table 1.

P10, line 241-254

*In considering negative consequences or harm associated with interventions we included criteria adapted from clinical guidelines and neurodiversity affirming practice. Specifically, we did not recommend:*

- *Interventions which focussed on the reduction of core features of autism are associated with harmful consequences and contradict current clinical guidelines [44, 45]. Core features include qualitative differences and impairments in reciprocal social interaction and social communication, restricted interests and activities, and rigid and repetitive behaviours [46].*
- *Interventions which contradicted current clinical guidelines [44, 46].*
- *Interventions associated with adverse events or adverse outcomes [43].*
- *Interventions which attempt to ‘cure, fix or normalise’ autistic people [2, 47] due to their negative impact upon quality of life [29].*
- *Interventions which target outcomes contradictory to the identified priorities of the autistic community [14, 24, 48-50].*

We have provided additional information regarding the role of autistic and non-autistic researchers within the research team.

P10, line 255-266

*The research team was made up of autistic and non-autistic professionals within speech and language therapy, psychology, psychiatry, occupational therapy, and teaching fields. Members of the team had research experience, and experience working with autistic people in clinical and education settings. As integrated members of the research team, autistic researchers contributed to the planning and design of this research study, and decision-making related to study outcomes alongside non-autistic colleagues. All team members held professional roles and contributed expertise to the study thus possibly reducing issues associated with power hierarchy sometimes found in autism research [51]. Arising disagreements concerning the classification of evidence were resolved through team discussion with reference to research recommendation classification (Table 1) and criteria regarding negative consequences or harm listed above. Inter-rater reliability was not recorded for this process.*

Additionally, we have provided reference to this within the study limitations.

P62, line 603-606

*Autistic researchers were integral to the research team this study and their views were not recorded separately from other research team members. Autistic research team members have professional backgrounds within research, health and education and are therefore not representative of all sections of the autistic community*.

6. **While Cohen’s Kappa (0.70) is acceptable it indicates there were a number of discrepancies, how were these handled?**

Author Response:

We have provided additional detail regarding the process for determining the outcome of arising discrepancies.

P10, line 263-266

*Arising disagreements regarding the classification of evidence were resolved through team discussion with reference to research recommendation classification (Table 1) and criteria regarding negative consequences or harm listed above until agreement was achieved. Inter-rater reliability was not recorded for this process.*

Results

7. **Table 3 has been expanded to provide additional information, however, there remains limited** **information provided about the study findings (effectiveness: positive, negative and/or mixed results AND negative consequences- not reported) to determine GRADE criteria. At present there is not enough information to verify the classifications (e.g., that no intervention meets criteria as evidence-based).**

Author Response:

Table 3 has been enhanced to include additional information on effectiveness of interventions, and negative consequences reported within the systematic reviews.

P50, line 383-387

*Column 3 of Table 4 outlines factors which may impact the acceptability of interventions to autistic adults including research limitations, indications of adverse effects, adverse outcomes, or priorities contradicting those identified by the autism community. Overall, results indicate a need for further robust research.*

We have also expanded the information included in Table 4 to provide additional detail regarding the effectiveness and acceptability of interventions.

These details are expanded throughout the sections describing the outcomes by type of intervention.

P56, line 397-483

***Pharmacological interventions***

*Five reviews [81, 88, 89, 98, 100] considered 139 studies evaluating pharmacological intervention for autistic individuals. One review was high quality (Table 3). Managing behaviours with medication as a first line of intervention or using medication including SSRIs (Selective Serotonin Reuptake Inhibitors) or Oxytocin for core features of Autism is not recommended (Table 4) [46, 100]. However, there was emerging evidence for use of medication as a last line of intervention. Oxytocin may offer some benefit but did not affect global clinical status [88]. Risperidone may be useful in the management of repetitive, aggressive, and self-injurious behaviour [81], although side-effects are problematic [98]. There was limited evidence to support the use of opioid antagonists to reduce self-injury in autistic adults [89]. However, fluoxetine or fluvoxamine may be useful in the management of repetitive and obsessive-compulsive behaviour and anxiety where other interventions are not available or possible due to the individual’s level of distress or aggression [98]. Overall, there is a need for future research to consider the acceptability of pharmacological interventions including further investigation of side-effects.*

***Employment focused interventions***

*Nine reviews of evidence for employment focussed interventions considered 100 unique publications [78, 80, 82, 83, 85-87, 90, 97]. None of the reviews were high quality (Table 3). Reviews revealed emerging evidence that supported employment including Individual Placement Support (IPS) and Project Search, yields positive outcomes for autistic people [78, 80, 82, 86, 87, 90, 97]. Notably, autistic adults, undertaking Project SEARCH with autism support were eleven times more likely to achieve employment than those attending special education [87]. However methodological concerns mean this result must be interpreted with caution as studies did not include comparable control groups or consider participant attrition [78]. Evidence for technology-supported interventions such as virtual reality training was unestablished as the relationship to paid employment was not confirmed [85]. Employment related social skills training research often focussed on alternative outcomes to employment status, such as interview skills performance, and therefore the evidence for such an approach is unestablished. Sheltered workshops were not recommended as they were not associated with supporting autistic people into employment but could provide other benefits. Further research is required to consider the impact of employment focussed interventions not only on employment status and wage, but also on quality of life [24].*

***Psychological therapies***

*There were 7 reviews of psychological therapies including 215 studies [74, 75, 79, 93-95, 99]. Only one review was of high quality (Table 3). The reviews revealed emerging evidence (Table 4) for the use of mindfulness for the reduction of self-reported depression symptoms in autistic adults without intellectual disability [71, 79, 84]. Studies provided emerging evidence for use of Cognitive remediation therapy to improve cognitive function, but small sample sizes and limited follow-up made it difficult to determine meaningful impact or maintenance of any benefit in the longer term [75].*

*There was unestablished evidence for the use of cognitive behavioural therapy (CBT), although small positive clinical effects on self-reported outcomes were observed [71, 99]. Within nine systematic reviews, which included CBT studies, 11 different types of CBT were described and included CBT combined with other interventions including behavioural techniques, mindfulness, and psychoeducation [93, 94].These major variations in the intervention provided meant it was not possible to conclude this intervention was effective. Additionally, there were expressed concerns regarding CBT which are outlined in table 4 and which should be considered in future research.*

*There was unestablished evidence for family therapy due to limited quality research [95] although non-randomised intervention studies suggest there may be improved knowledge and understanding of core disorder (ASD), and coping styles post-intervention [95]. Acceptance and Commitment Therapy was not recommended due to limited research and insufficient rigour [74] to suggest ACT is effective in the management of psychological distress for individuals with ID [74].*

***Mixed interventions and approaches***

*Twelve systematic reviews considered 300 studies within 11 sub-categories of intervention identified [20, 71-73, 76, 77, 83, 84, 91, 92, 96, 101]. Two reviews were rated as high quality (Table 3). Evidence for most of interventions in this grouping was unestablished or not recommended (Table 4). However, there was emerging evidence for the use of the PEERS programme in reducing social anxiety and loneliness [20]. Although, concerns regarding social skills interventions including PEERS includes the risk they teach camouflaging which has been associated with suicidality [71, 103]. There was also emerging evidence for social cognitive interventions [73, 83] although there is a need to explore whether these interventions are required by the autism community. There was unestablished evidence for social skills interventions [20, 77, 83, 84, 92, 101] and studies were criticised for their limited input from autistic people instead relying on parent or caregiver reports [20], Results indicate positive effects from communication interventions [20, 83, 101] but there is a need for additional robust research. Music, and dance therapies research did not demonstrate the effectiveness of this type of intervention [72, 83]. Music therapy intervention studies suggest this may have a positive impact on autistic children regarding social interaction and communication, although studies did not find significant difference in symptom severity [72]. These studies relied heavily on diagnostic measures to analyse social or behavioural differences before and after intervention [72]. Diagnostic measures are generally insensitive to change and indicate a focus on the treatment of core symptoms. There were no randomised controlled trials investigating the impact of music therapy. There is also a need for further robust research investigating the benefits of environmental, leisure and participation focussed interventions [20, 71, 73, 76, 83]. Evidence for behaviourist approaches was unestablished [71, 73, 76, 101]. Although, the autistic community have expressed concerns regarding the use of applied behaviour analysis and further engagement is required to determine whether these approaches should be used [21]. Electro-convulsive therapy was not recommended, and autistic researchers involved in the systematic review did not feel this was an appropriate intervention for autistic people [71]. There is evidence of negative responses to this intervention and of high risk of bias in research studies [71]. Major concerns were expressed about this intervention including possible damage to brain and memory [71]. Auditory Integration Training was not recommended due to absence of evidence, and safety concerns [91]. Group academic and social skills training [84] were not recommended. Group social skills interventions were more effective for enhancing knowledge and understanding, rather than increasing specific social skills [92].*
